# Gestational exposure to metformin programs improved glucose tolerance and insulin secretion in adult male mouse offspring

**DOI:** 10.1038/s41598-018-23965-4

**Published:** 2018-04-10

**Authors:** Brigid E. Gregg, Nathalie Botezatu, Joshua D. Brill, Hannah Hafner, Suryakiran Vadrevu, Leslie S. Satin, Emilyn U. Alejandro, Ernesto Bernal-Mizrachi

**Affiliations:** 1 Department of Pediatrics, Division of Diabetes, Endocrinology and Metabolism, University of Michigan Medicine, Ann Arbor, USA; 2 Department of Pharmacology, Brehm Center for Diabetes Research, University of Michigan, Ann Arbor, USA; 3 Department of Integrative Biology and Physiology, University of Minnesota Medical School, Minneapolis, USA; 4 Division of Endocrinology, Diabetes and Metabolism, University of Miami Miller School of Medicine, Miami, USA

## Abstract

Pancreatic β-cells are exquisitely sensitive to developmental nutrient stressors, and alterations in nutrient sensing pathways may underlie changes observed in these models. Here we developed a mouse model of *in utero* exposure to the anti-diabetic agent metformin. We have previously shown that this exposure increases offspring pancreatic β-cell mass at birth. We hypothesized that adult offspring would have improved metabolic parameters as a long-term outcome of metformin exposure. Virgin dams were given 5 mg/mL metformin in their water from E0.5 to delivery at E18.5. Body weight, glucose tolerance, insulin tolerance and glucose stimulated insulin secretion were analyzed in the offspring. When male offspring of dams given metformin during gestation were tested as adults they had improved glucose tolerance and enhanced insulin secretion *in vivo* as did their islets *in vitro*. Enhanced insulin secretion was accompanied by changes in intracellular free calcium responses to glucose and potassium chloride, possibly mediated by increased L channel expression. Female offspring exhibited improved glucose tolerance at advanced ages. In conclusion, in this model *in utero* metformin exposure leads to improved offspring metabolism in a gender-specific manner. These findings suggest that metformin applied during gestation may be an option for reprogramming metabolism in at risk groups.

## Introduction

Metformin is the most widely prescribed oral agent for the treatment of diabetes in the United States^1^. In addition, metformin is used for the treatment of gestational diabetes in some countries and is currently under study for this application in the United States^2^. Metformin has also been used to improve fertility and decrease miscarriage rates in patients with polycystic ovary syndrome^3^. Despite its widespread use, the impact of metformin exposure during pregnancy on the metabolic status and glucose homeostasis of offspring has not been studied in detail.

The developing pancreas is exquisitely sensitive to nutrient signals, and nutrient sensors are thought to play a role in the alterations observed in β-cells that are subjected to nutrient stressors during development^4^. One such nutrient sensor, AMP-activated protein kinase (AMPK) is a molecular target of metformin. While not all of the intracellular effects of metformin are understood, treatment of cells with metformin leads to an alteration in the cellular ATP/ADP ratio, which in turn activates AMPK^5,6^. The implication for alterations in the AMPK pathway on developing β-cells has not been studied in detail. There are few *in vivo* models of AMPK overexpression and alterations in this pathway do not appear to impact early β-cell lineage. Treatment of cells with metformin also has direct and AMPK-independent effects on the mTOR signaling pathway^7^. The intricacies of the animal AMPK models make comparisons between existing models and the present work difficult.

We have previously shown that metformin exposure during gestation increases the number of pancreatic (Pdx1) and endocrine (Ngn3) cell progenitors^8^. In addition, the islets of offspring who were exposed to metformin have an increased fraction of β-cells at birth. The implications of the changes induced by this experimental system for the β-cell morphology and function of the adult offspring have not been reported previously. Other reports of metformin-induced programming do exist but employed different doses and routes of metformin administration^9–11^. However, the effects of metformin on pancreatic β-cell outcomes have not been explored in detail. We thus sought to examine the impact of gestational metformin exposure on the metabolic health of adult offspring and to determine the mechanisms that underlie the changes we observed.

## Materials and Methods

### Metformin programming mouse model

The details of the *in utero* metformin exposure model have been published previously^8^. Briefly, we purchased 8 week-old virgin C57Bl6 animals from Jackson Laboratories and adapted to control diet (D02041001B, Research Diets Inc., New Brunswick, NJ, USA) for 3 weeks. Upon vaginal plug detection females were given unadulterated water or sterile water with metformin (Sigma-Aldrich, St Louis, MO) at 5 mg/mL. Water was changed weekly and the treatment was continued until birth. At that point mothers resumed the filtered water supply in the animal room. All the studies were approved by the University of Michigan Institutional Care and Use Committee and performed in accordance with their guidelines under an approved animal protocol.

### Pancreas collection

We harvested adult pancreata at 3 months of age from C57Bl6 metformin and control animals. We then fixed pancreata in 3.7% formalin in PBS overnight.

### Primary mouse islet isolation

A collagenase digestion technique was used to isolate pancreatic islets^12^. The common bile duct was perfused with 1 mg/ml collagenase XI (Sigma-Aldrich, St. Louis, MO) in HBSS (Life Technologies, Carlsbad, CA). Pancreata were digested at 37 °C for 15 min, and then cold HBSS with 2.5% FBS (Life Technologies) was added. After centrifugation at 1500 rpm for 2 min, the pellet was washed 3 times with HBSS with 2.5% FBS. A histopaque gradient was then set up and pancreata were centrifuged at 2000 rpm at 10 degrees Celsius for 20 minutes. The interphase layer was collected, filtered through a 70 µm cell strainer (BD Falcon, BD Biosciences, San Diego, CA) and rinsed with HBSS. The islets were plated in RPMI 1640 with 5 mM Glucose, 10% FBS, 100 IU/ml penicillin, 100 g/ml streptomycin. The islets recovered overnight in this media before experiments were carried out.

### Primary mouse islet *in vitro* insulin secretion assay

After recovering overnight isolated islets were cultured in Krebs-Ringer buffer with HEPES (KRBH) with 2 mM glucose for 60 minutes before placing in 22 mM glucose for 30 minutes. To assess maximum insulin secretory capacity, we then placed islets in KRBH with 2 mM glucose and 30 mM KCl for 30 min. Insulin levels were measured with ELISA (Mouse-Insulin Ultrasensitive, ELISA, ALPCO Immunoassays, Salem, NH).

### Immunofluorescent staining and islet morphology analysis

After formalin fixation, pancreatic tissues were embedded in paraffin using standard techniques. 5 µm thick sections were cut and then deparaffinized, rehydrated and blocked as previously described^13^. Sections were incubated overnight at 4 °C with antibodies to the following proteins: insulin (anti-guinea pig, Dako, Copenhagen, Denmark), Ki67, Phosphorylated S6 (Ser240, Cell Signaling, Danvers, MA). Secondary antibodies conjugated to FITC or Cy3 (Jackson Immunoresearch, West Grove, PA) were then applied the following morning. DAPI-containing mounting media (Vector Laboratories, Burlingame, CA) was applied prior to adding coverslips. Using five insulin-stained sections (5 µm) separated by 200 µm (adult pancreas) per pancreas, total pancreas and insulin-positive cell areas were measured using Image Pro Software (Media Cybernetics, Inc., Bethesda, MD). β-cell mass (calculated as the average β-cell fraction multiplied by pancreas weight) assessment was performed using Objective Imaging’s Surveyor automated scanning with a Leica fluorescent microscope (Leica Microsystems, Wetziar, Germany). Proliferating cells were identified as those co-staining with monoclonal rabbit anti-Ki67 and insulin antibodies. At least 1000–3000 stained cells from each treatment were counted for the analysis.

### Protein and RNA Isolation

Protein and RNA samples were prepared from three to four islet preparations from the control (ctrl) and metformin (met) treatment groups. Samples were rinsed with PBS and then incubated in lysis buffer (Cell Signaling, Danvers, MA) or with trizol (Sigma-Aldrich). Samples were flash frozen with liquid nitrogen and stored at −80 °C until homogenization. For gene expression analysis RNA was isolated using trizol in a commercially available kit with columns (Qiagen, Valencia, CA).

### Quantitative real-time PCR for gene expression

RNA for gene expression analysis was isolated using RNeasy (Qiagen, Valencia, CA), as above. cDNA synthesis was performed using random hexamers and reverse transcription was performed using Superscript II (Life Technologies, Carlsbad, CA) according to the manufacturer’s protocol. Real-time PCR was performed on an ABI 7000 sequence detection system using SYBR Green (Applied Biosystems, Foster City, CA) and primers purchased from Applied Biosystems (Foster City, CA)(Supp. Table 1).

### Protein quantifications

Immunoblotting was performed as previously described^14^. Briefly, after treatment with PBS islets were washed and then cell lysis buffer (Cell Signaling, Danvers, MA) with protease inhibitor cocktail and phosSTOP tablets from Roche Applied Science (Indianapolis, IN) was added. Primary antibodies against ACC, phosphorylated S6 (Ser 240 and 235), Akt (S473), SNAP 25 and total Akt were purchased from Cell Signaling (Danvers, MA, USA). An antibody against CSP-α was provided by Synaptic Systems (Gottingen, Germany). A mouse β-actin antibody was purchased from Sigma-Aldrich. Densitometry analysis was performed using Image J (NIH, Bethesda, MD).

### Glucose and insulin tolerance tests

Glucose tolerance tests were performed using intraperitoneal injection of 2 g/kg glucose after a 15 hour fast. Insulin tolerance tests were performed with an intraperitoneal injection of 0.75 Unit/kg insulin (Novolin, Novo Nordisk Inc., Princeton, NJ) after a 6 hour fast. Blood glucose was measured using AccuChek Aviva glucometer (Abbott, Alameda, CA) for 2 hours after glucose delivery and 90 minutes after insulin delivery. Fasting glucose and insulin were measured using tail vein blood after overnight fasting. Plasma insulin levels were measured using mouse ultrasensitive insulin ELISA kit (ALPCO, Salem, NH).

### Measurement of islet insulin content

To measure islet insulin content 10 size-matched islets were placed into a 1.5 ml tube containing 100 µl cell lysis buffer (Cell Signaling, Danvers, MA). At the time of the ELISA samples were diluted with Krebs-Ringer buffer 1:2500 and insulin concentration was measured using the Mouse Ultrasensitive Insulin ELISA kit (ALPCO, Salem, NH). Insulin levels were corrected to total DNA content of the sample, as measured by Qubit Fluorometer (Life Technologies, Carlsbad, CA).

### Measurements of intracellular Ca^2+^

Islets were preloaded with 2.5 μM Fura2-AM (Thermo-Fisher) in 0.1% DMSO for 30 min at 37 °C and washed for 10 minutes before recording. The recording solution contained, in mM: 145 NaCl, 5 KCl, 1.2 MgCl_2_, 2.6 CaCl_2_, 10 HEPES, and glucose as indicated; pH 7.4. Imaging parameters were identical to those described in Merrins *et al*.^15^ and relative Ca^2+^ is reported as the excitation ratio R340/380, with emission collected at 502 nm.

### Statistical analysis

Statistical significance was assessed through the Mann-Whitney test (u-test) or t-test where appropriate using GraphPad Prism (version 6.0c, GraphPad Software, La Jolla, CA, USA). Results are presented as mean values +/− standard error of the mean. Significant results were those with a p-value < 0.05.

## Results

### Adult offspring exposed to intrauterine metformin showed no differences in their baseline metabolic parameters

**Figure 1:**
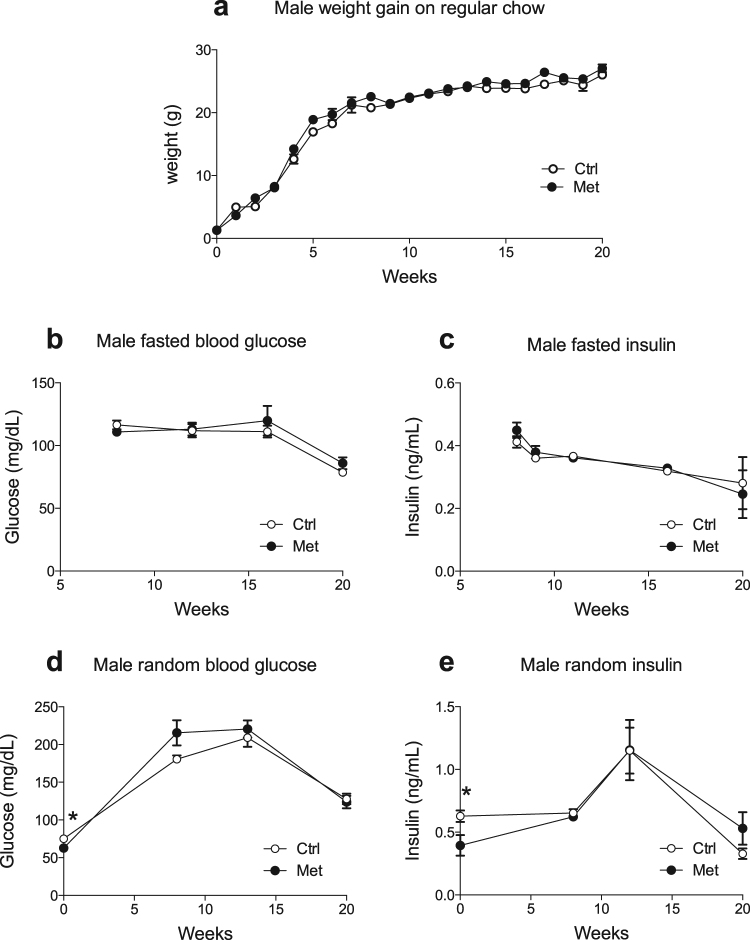
Metformin in utero does not alter weight gain, glucose or insulin levels in males. Male offspring of dams exposed to metformin (•) as compared to control offspring (). Weight gain was tracked weekly (**a**) (n = 16–20). Fasted insulin and glucose levels (**b** and **c**) were collected every 4–6 weeks (n = 6–13). Random glucose and insulin levels were collected at birth and then every 4–6 weeks (**d** and **e**) (n = 6–13).

In order to determine the programming effects of metformin exposure during gestation on offspring metabolic parameters were assessed in young adult mice up to the age of 20 weeks. Offspring of both sexes had decreased random blood glucose at birth, as shown previously and in Fig. 1d and Supp. Figure 1D^8^. In the present study the offspring of both sexes also had decreased random insulin on the day of birth (Fig. 1e, Supp. Figure 1E). Thereafter, male offspring exhibited no differences in body weight, fasting blood glucose, fasting insulin, fed blood glucose or fed insulin compared to controls at up to 20 weeks of age (Fig. 1). Females likewise had no differences in body weight, glucose or insulin when they were on a regular chow diet (Supp. Figure 1). Finally, AMPK activity in the liver assessed by phosphorylation of Acetyl-CoA carboxylase were similar indicating that metformin administration during pregnancy has no permanent effects on AMPK signaling in the liver of the offspring (data not shown).

### Adult male and older female offspring exposed to metformin during fetal life exhibited improved glucose tolerance and conserved insulin sensitivity

**Figure 2:**
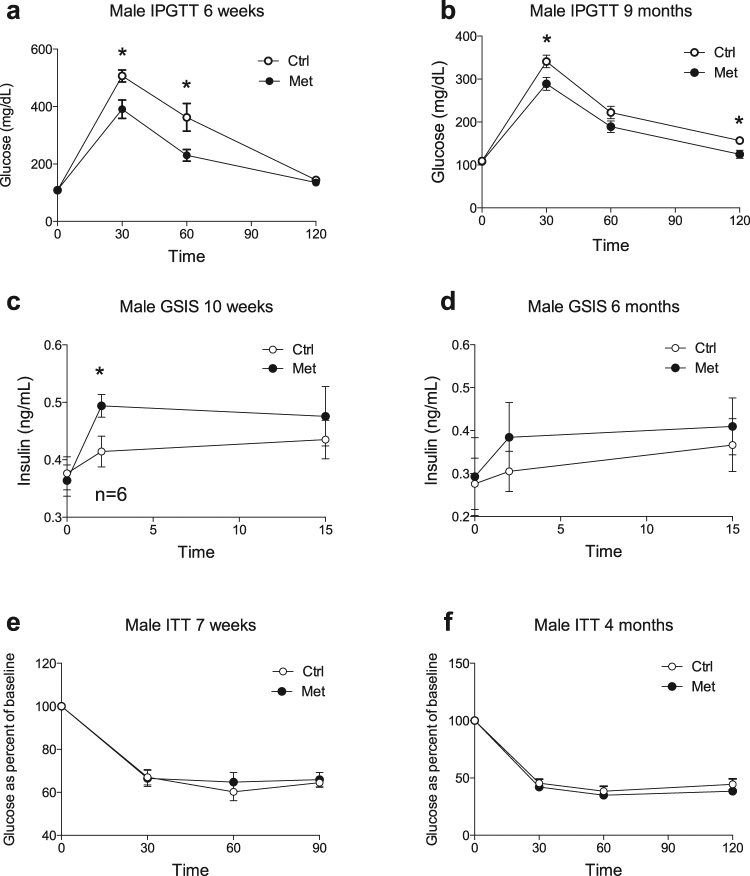
Male offspring from metformin exposed dams have improved glucose tolerance and insulin secretion *in vivo*. Male offspring of dams exposed to metformin (•) as compared to control offspring (). (**a**,**b**) Intraperitoneal glucose tolerance testing in 6 week (**A**) (n = 12) and 9 month (n = 9) (**b**) old males. (**c**,**d**) *In vivo* GSIS in 10 week (**c**) and 6 month (**d**) old males (n = 7). (**e**,**f**) Insulin tolerance testing in 7 week (**e**) (n = 6) and 4 month (**f**) old males (n = 6) *p < 0.05.

**Figure 3:**
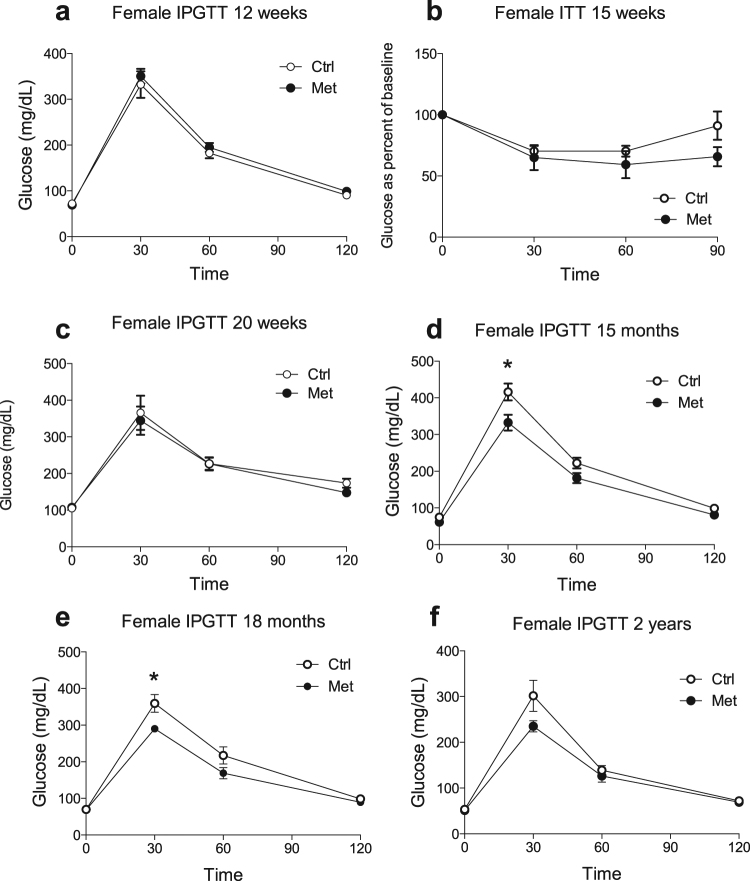
Female offspring have improved glucose tolerance late in life. Female offspring of dams exposed to metformin (•) as compared to control offspring (). Intraperitoneal glucose tolerance testing in female offspring of metformin exposed dams versus controls throughout their lifespan (**a**,**c**–**f**). N numbers for A = 10, C = 5–7, D = 4–9, E = 6–11 and F = 4. Insulin tolerance testing at 15 weeks of age (**b**) (n = 5–6). *p < 0.05.

We next performed *in vivo* metabolic testing in order to examine the metabolic responses of offspring. Six week-old male offspring exposed to metformin had improved glucose tolerance in response to an intraperitoneal glucose injection (Fig. 2a). In addition, they secreted more insulin in response to a glucose stimulus *in vivo* (Fig. 2c). Changes in glucose tolerance and insulin secretion observed in male met offspring became less pronounced over time but still persisted into later adulthood (Fig. 2b,d). When their insulin sensitivity was assessed by intraperitoneal insulin injection no differences were found between the groups (Fig. 2e,f). Female offspring exposed to metformin *in utero* had similar intraperitoneal glucose and insulin sensitivity to controls (Fig. 3a–c). However, IPGTT performed in aged females (1–2 years of age) who were exposed to metformin *in utero* showed improved glucose tolerance compared to controls (Fig. d–f).

### Male and female metformin treated offspring showed no changes in β-cell mass

**Figure 4:**
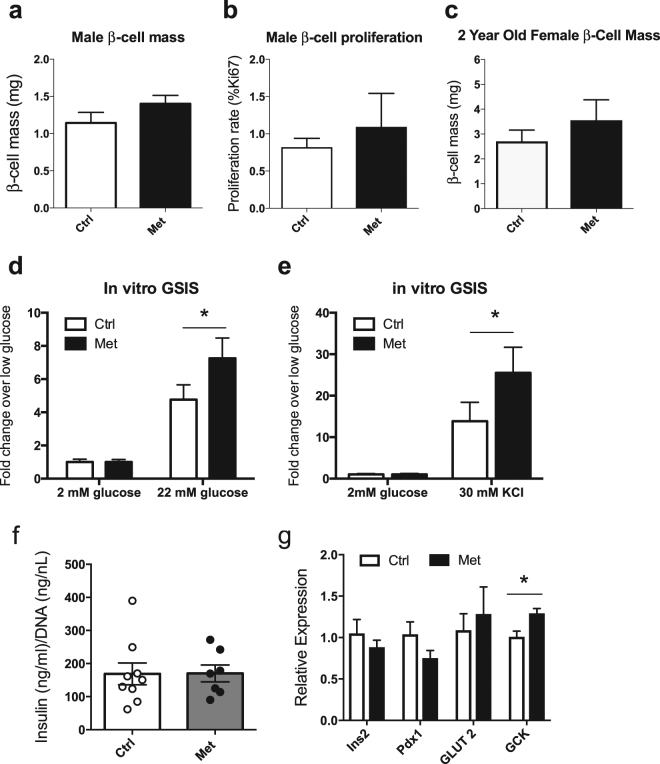
Male Met offspring have improved insulin secretory responses *in vitro*. Pancreas morphology in 3 month old offspring of metformin treated or control dams examining β-cell mass (**a**) (n = 5) and β-cell proliferation (**b**) (n = 3). β-cell mass at 2 years in female offspring n = 4 per group (**c**). (**d**–**e**) *In vitro* islet secretion assay in response to high glucose (**d**) and potassium chloride (**e**) (n = 6). (**f**) Islet content from 10 representative islets and normalized to DNA content from n = 7–9 animals per group. Relative expression of Ins2, Pdx1, GLUT2 and GCK mRNA from n = 4–5 animals per group (**g**).

To understand the cause of the improved insulin secretion we observed in adult male offspring who were exposed to metformin we next examined the morphology of their pancreata to determine β-cell mass. As mentioned previously, newborn offspring of dams (either males or females) exposed to metformin exhibited an increase in pancreatic β-cell fraction at birth^8^. When pancreata from male offspring were examined at 3 months, however, β-cell mass was not found to be significantly different in the *in utero* treated offspring versus controls (Fig. 4a). Likewise, we saw no difference in β-cell proliferation rates between the two groups (Fig. 4b). β-cell mass in 2 year old female offspring was also not different between the groups (Fig. 4c).

### Met offspring islets show increased insulin secretion *in vitro*

Since there were no changes in β-cell mass to explain the improved insulin secretion seen *in vivo*, we examined the effect of gestational exposure to metformin on islet insulin secretion *in vitro* by examining responses to insulin secretagogues. No differences in insulin secretion were observed at low glucose concentrations (Fig. 4d). However, islets isolated from adult male offspring showed increased insulin secretion in response to high glucose (22 mM) or KCl (30 mM) (Fig. 4d,e). Isolated islets from male offspring exposed to metformin also did not differ in their insulin content from controls and showed no difference in Ins2 mRNA as assessed by qPCR (Fig. 4f,g). Due to the increase in insulin secretion we observed in response to high glucose, expression levels were also assessed for GLUT2 (*SLC2A2*) and glucokinase (*GCK*). While there was no significant difference in GLUT2 expression, the metformin group showed a mild but significant increase in glucokinase mRNA.

### Metformin programmed islets showed no changes in candidate exocytotic proteins

**Figure 5:**
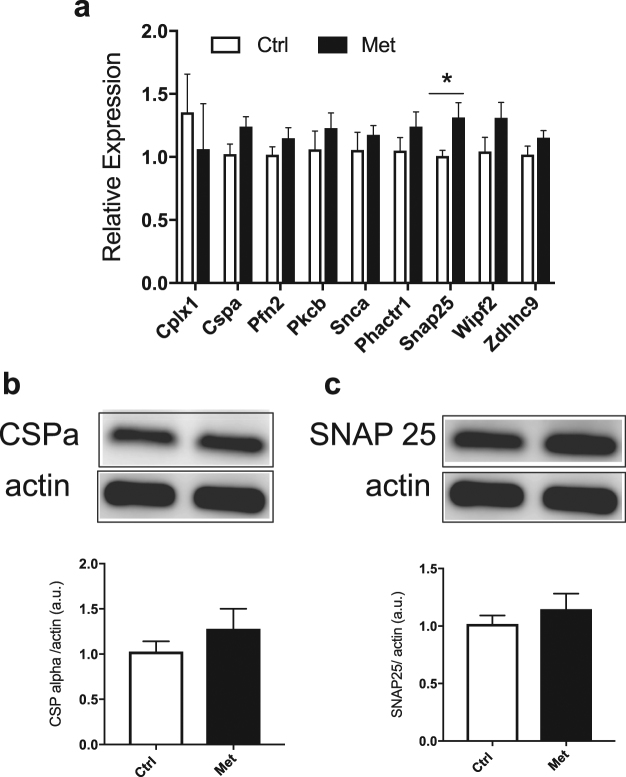
Male offspring from metformin exposed dams have few changes in expression or protein levels of candidates in the granule exocytosis pathway. (**a**) Expression of candidate granule exocytosis machinery from islets isolated from 4–5 month old animals, n = 9. (**b**) A representative western blot of CSPα and quantification of CSPα corrected to β-actin from male islets. (**c**) A representative western blot and quantification for SNAP25. The representative western blot images are selected from 2 adjacent wells of a larger western blot shown in Supplemental Fig. 2. These experiments represent n = 9 animals run in 3 separate experiments.

Given the improvement in insulin secretion we observed in response to membrane depolarization by KCl, we went on to examine factors important for β-cell stimulus-secretion coupling. In models of developmental programming epigenetic alterations often underlie the observed phenotypes^16^. Due to the changes in insulin secretion we observed in our model, we assayed the levels of candidate genes identified in previous studies that studied epigenetic alterations of genes involved in secretory responses^17^. We focused on candidate members of the exocytotic machinery. Examination of these by quantitative PCR showed no differences in CSP-α (*DNAJC5*) or PCK-β (*PRKCB*), however there was an increase in SNAP-25 (*SNP25*) mRNA in the metformin group (Fig. 5a). We went on to examine changes at the protein level for candidates for which there were commercially available antibodies. We performed islet immunoblots for CSP-α and SNAP-25 and found no difference in the levels of these proteins in the metformin group when compared to the controls (Fig. 5b,c).

### Metformin programmed islets exhibited increased free calcium entry in response to insulin secretagogues

**Figure 6:**
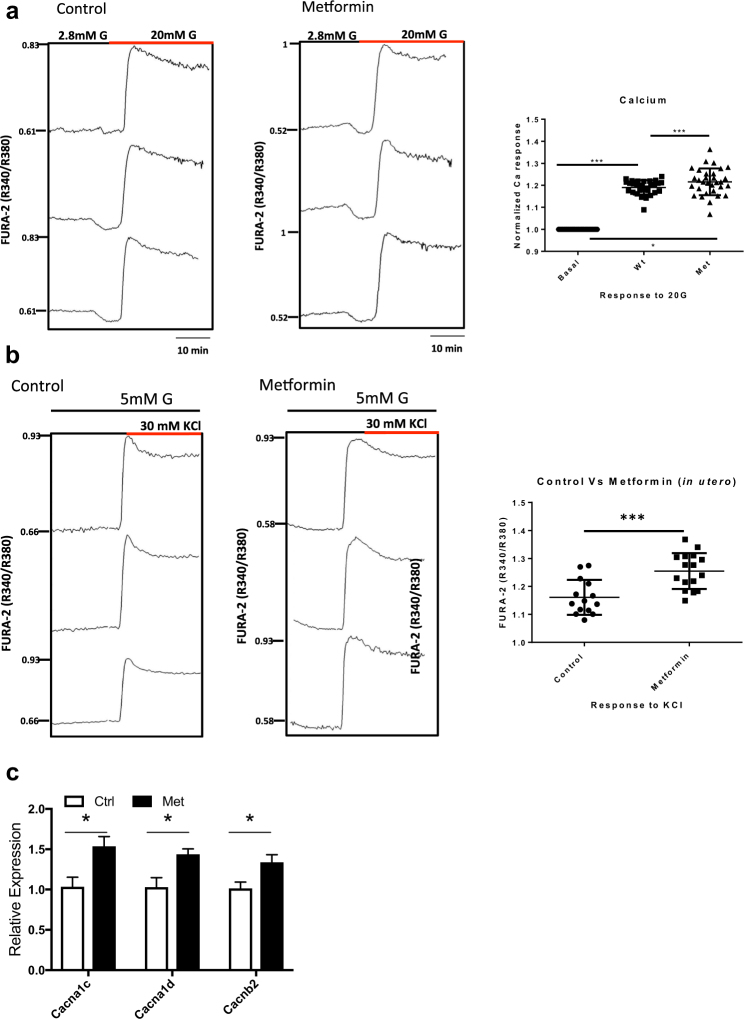
Increased islet calcium entry in male offspring from metformin exposed dams. Calcium entry as measured by FURA-2 imaging. A representative tracing of control (■) versus metformin (▲) islets in response to high glucose with a quantification of the calcium response (**a**). Representative tracings and calcium quantification for responses to 30 mM potassium chloride (**b**) for control (●) versus metformin (■) islets. These tracings represent n = 6 animals per group run on 3 separate experiments. (**c**) Expression of calcium channel subunits from islets isolated from 4–5 month old animals, n = 9 ***p < 0.001, *p < 0.05.

We next explored alternative processes that could explain the improved insulin secretory response to gestational exposure to metformin, since exocytosis candidate genes were unchanged by the treatment. Intracellular free calcium was measured in islets from adult male offspring from treated versus control dams using FURA-2 dye. When the islets were exposed to high glucose the Ca^2+^ responses of metformin exposed islets were significantly larger (Fig. 6a). Similarly, islets from offspring exposed to metformin showed greater Ca^2+^ responses to KCl (Fig. 6b). These results indicate that the enhanced insulin secretion that we observed in islets from metformin-exposed offspring was due to potentiated Ca^2+^ entry into the β-cell. In order to understand whether the potentiated Ca^2+^ entry we observed might reflect increased expression of L-type calcium channels, we probed treated vs. control islets for Ca^2+^ channel RNA transcripts. As shown, we found increased mRNA for CaV1.2 (CaCna1c) and CaV1.3 (CaCna1d), which are L-type calcium channel alpha subunits, and increased beta-2 subunit (CaCnb2) message in the metformin versus control groups (Fig. 6c). This suggests that metformin exposure may act, at least in part through epigenetic modification of L type Ca^2+^ channel transcription.

## Discussion

In this study we sought to extend the observations from our previous work demonstrating an increase in β-cell mass at birth after metformin programming. In this study we found that the increase in β-cell mass did not persist into adulthood. This finding may be explained by the differing factors that regulate developmental versus adult β-cell proliferation^18^. In this experimental system the withdrawal of metformin at birth may have impacted the postnatal proliferative rate. When the islets were studied in isolation, male offspring from dams given metformin *in utero* had improved glucose tolerance because of increased stimulated islet insulin secretion. Female offspring did not manifest a metabolic phenotype until they reached an advanced age. This sexual dimorphism raises the possibility of a sex-steroid dependence of the phenotype. Indeed sex steroids have been shown to play important roles in β-cell insulin secretion^19^. Underlying the improvement in insulin secretion was an increase in islet Ca^2+^ entry in response to either glucose or KCl. This suggests that potentiated Ca^2+^ channel activity (e.g. possibly secondary to increased cyclic AMP/PKA activity) was a major contributor to the phenotype. The contribution of the increase in glucokinase expression is interesting and suggests that this could result in increase in glycolytic flux. Future experiments could be designed to validate these results by examining enzyme activity and assess if the increase expression is a consequence of transcriptional control by epigenetic modifications induced by metformin exposure in utero. Glucokinase expression was found to be reduced in a rat model of developmental programming from *in utero* low protein diet, confirming that this enzyme may be susceptible to epigenetic modification^20^.

The novel findings in glucose homeostasis and insulin secretion observed by gestational metformin in this model are in contrast to previous published studies. The work of Salomaki *et al*. uses a similar experimental design with metformin during gestation only, however their exposure led to no change in glucose tolerance while animals were on a normal chow diet^9^. Potential explanations for the differences between these studies and our current findings include the stress of gavage administration of metformin and the lower blood metformin levels achieved using gavage as compared to the present study^9^. The metformin levels achieved in our study would be consistent with what would be expected on a typical dose of metformin in pregnancy (equivalent of 2000 mg daily)^21^. Finally, the effects of gestational metformin on β-cell measurements were not determined in the in the study by Salomaki *et al*. Other models are difficult to compare to the present study as they overlay metformin treatment on gestational high fat diet exposure or extend the treatment period up to weaning^10,22^.

Human clinical studies that follow the offspring of women treated with metformin during pregnancy have been published. In a large cohort follow-up of women treated with metformin versus insulin for gestational diabetes examination of children at age 2 revealed a difference in skinfold thickness on caliper measures but no change in body composition on DEXA scanning of a smaller subset^23^. That study did not publish parameters of glucose homeostasis. In another study of offspring of women treated with metformin for polycystic ovary syndrome in pregnancy there were no consistent changes in growth trajectory in the children to age 5 years^3^. Parameters of glucose homeostasis were also not studied in this cohort. This same group went on to examine a larger group of offspring from a multi-center trial and found that the infants had increased weight at age 1 after *in utero* metformin exposure^24^.

Our findings of increased intracellular calcium responses by gestational metformin are reminiscent of previous studies in a sheep model of placental restriction^25^. In this work the adverse intrauterine environment led to a change in the expression of the voltage gated L-type calcium channel α1D, which was proposed to lead to impaired β-cell function^25^. Models of programmed increases in islet calcium entry have not been previously reported. Indeed, when we examined the expression of L-type Ca^2+^ channels in pancreatic islets in our experimental model we found an increase in the metformin-treated offspring group. It remains to be determined if epigenetic changes underlie this expression difference, as was reported for L-type calcium channels in hypertensive rat arteries. In this model, L-type calcium channel expression was regulated by promoter methylation and the expression of a specific micro RNA species was altered^26^. Future studies could be designed to test this hypothesis in islets from mice exposed to metformin *in utero*.

While studies of nutritional and toxin stressors causing impairment in β-cell mass and/or function are extensively reported, fewer studies exist to demonstrate a potential intervention to improve this outcome. Groups have examined the potential for leptin, GLP-1 and statin treatments to improve the outcome of β-cell stressor environments using animal models^27–29^. While this study did not examine an *in utero* stressor, the results here indicate that gestational metformin treatment could have a beneficial effect on offspring glucose homeostasis. Indeed, groups have published the ability of metformin treatment along with high fat diet during gestation and/or lactation to improve glucose tolerance in adult high fat diet offspring^22,30^. Despite these appealing results from animal studies, the role of metformin in human fetal life remains unclear. Human studies of the lasting effects on metabolism in offspring of women treated with metformin will continue to provide insight and help clarify what we have observed using animal models.

## Electronic supplementary material

Supplementary Figures
