# Supplementary material for: Gestational exposure to metformin programs improved glucose tolerance and insulin secretion in adult male mouse offspring
Source: Sci Rep. 2018 Apr 10;8:5745. doi: 10.1038/s41598-018-23965-4 (PMC5893596; doi:10.1038/s41598-018-23965-4)
Supplement: Supplementary file 1 — Supplementary Figures [file 41598_2018_23965_MOESM1_ESM.pdf]

Gestational exposure to metformin programs improved glucose tolerance and insulin secretion in adult male mouse offspring

Brigid Gregg, Nathalie Botezatu, Josh Brill, Hannah Hafner, Suryakiran Vadrevu, Leslie Satin, Emilyn Alejandro, Ernesto Bernal-Mizrachi

Supplemental Figure 1. **Metformin in utero does not alter weight gain, glucose or insulin levels in females.**
Female offspring of dams exposed to metformin (●) as compared to control offspring (❍). Female offspring of dams exposed to metformin (black circles) as compared to control offspring (open circles). Weight gain was tracked weekly (A). Fasted and fed insulin levels (B and C) were collected every 6-8 weeks, n=8-11. Random glucose and insulin levels were collected at birth and then every 4-6 weeks (D and E), n=6-15.

Supplemental Figure 2. **Male offspring from metformin exposed dams have no changes in expression or protein levels of candidates in the granule exocytosis pathway.**
Full length western blots from which the images for Figure 5 were selected. The blots were done in sequence and the images appear in the order they were blotted.

Supplemental Table 1. **Real time PCR primer sequences.**

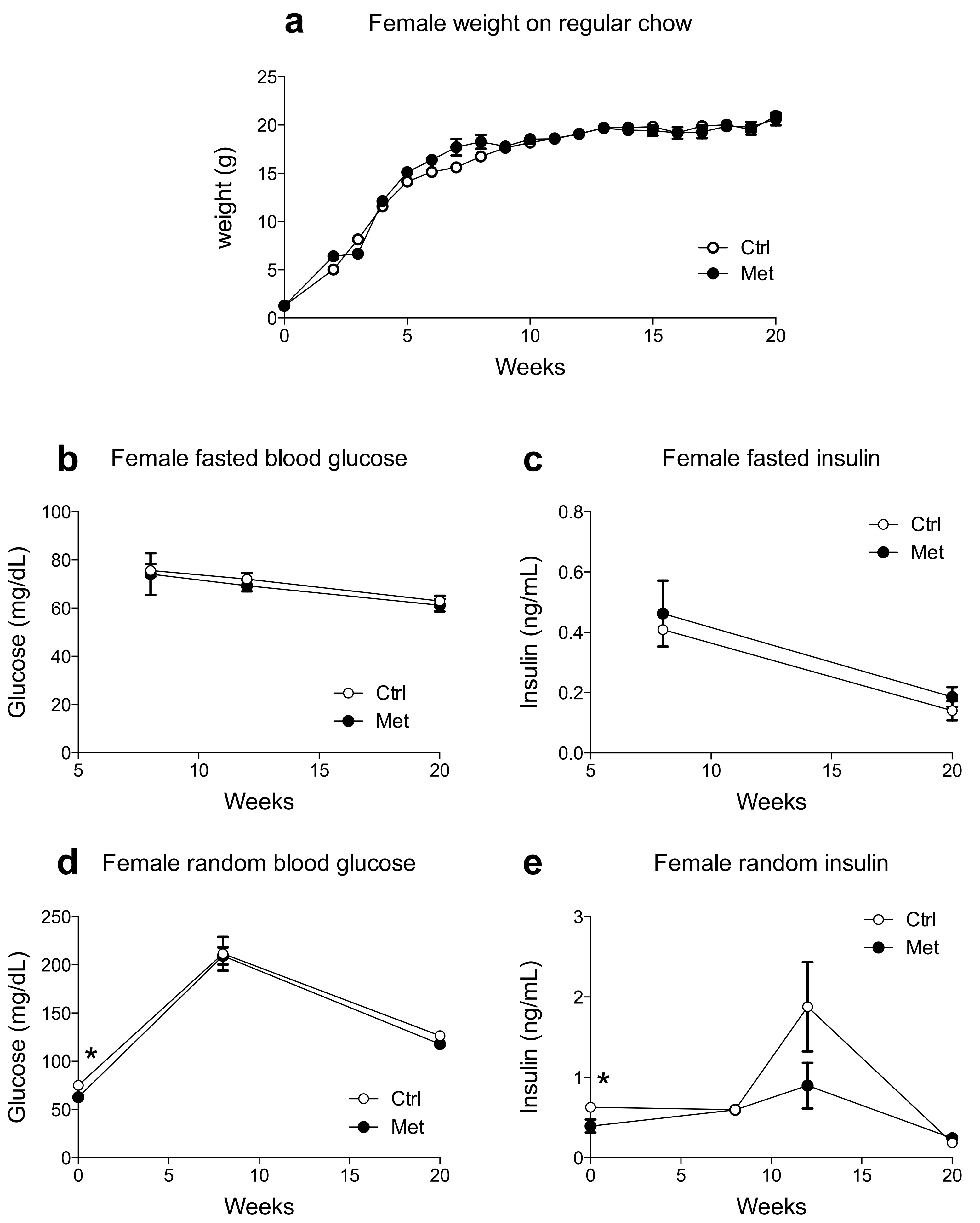
a
Female weight on regular chow
weight (g)
25
20
15
10
5
0
0
5
10
15
20
Weeks
Ctrl
Met
b
Female fasted blood glucose
Glucose (mg/dL)
100
80
60
40
20
0
5
10
15
20
Weeks
Ctrl
Met
c
Female fasted insulin
Insulin (ng/mL)
0.8
0.6
0.4
0.2
0.0
5
10
15
20
Weeks
Ctrl
Met
d
Female random blood glucose
Glucose (mg/dL)
250
200
150
100
50
0
0
5
10
15
20
Weeks
*
Ctrl
Met
e
Female random insulin
Insulin (ng/mL)
3
2
1
0
0
5
10
15
20
Weeks
*
Ctrl
Met

Supplemental Figure 2

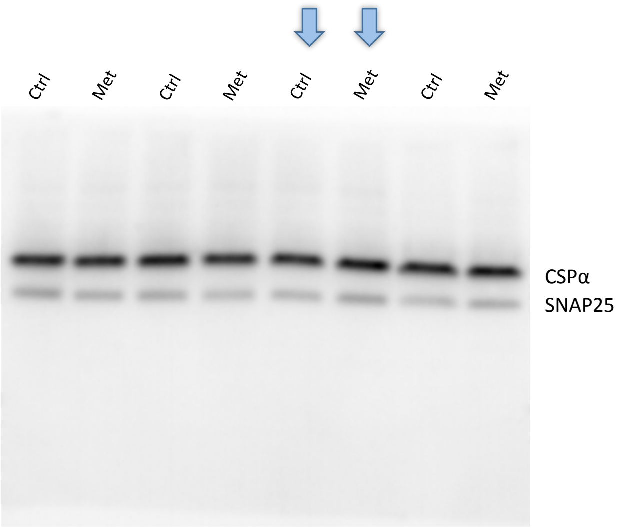

1. CSP antibody was blotted and read second (SNAP25 was blotted first which is visible here, but a reblot was used for final analysis)

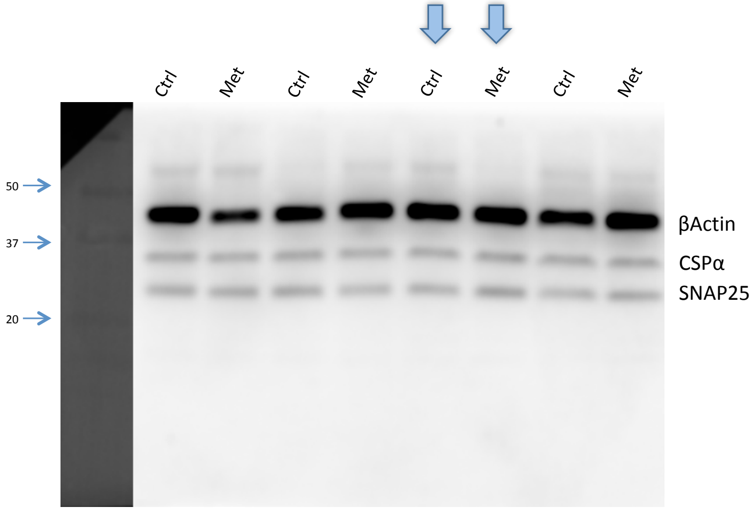

2. βActin antibody was blotted and read third (SNAP25 and CSP are visible from previous blots)

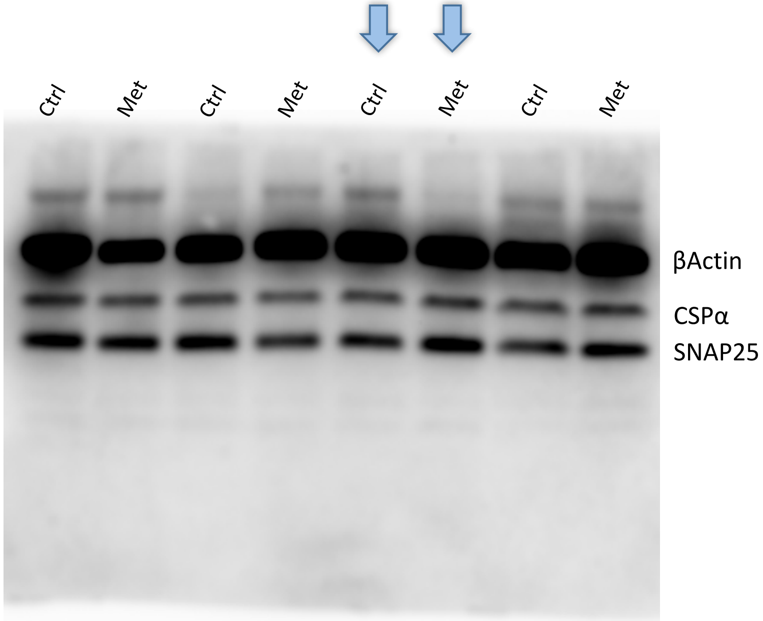

3. SNAP25 antibody was re-blotted (βActin and CSPα are visible from previous blots)

Supplemental Table 1.

Gene	Forward (5'-3')	Reverse (5'-3')
GLUT2	TCA GAA GAC AAG ATC ACC GGA	GCT GGT GTG ACT GTA AGT GGG
Gck	CAT CAG GAG GCC AGT GTA AAG	TCC CAG GTC TAA GGA GAG AAA G
Cacna1c	GAC CAT CGG GAA CAT CGT AAT	ATT CTG CCT CCG TCT GTT TAG
Cacna1d	CGT TGG TCC TGT CTA CAA CTA C	GAT GAC GAA GCC CAC AAA GA
Cacnb2	CAA CAG AGT GGC TCC AGA AA	CAG GTC AGG TAT CTG GGT ATT G
Cplx1	CCA TGG AGT TCG TGA TGA AA	CCA AGC ATC TTC CCC ATG T
CSPa	CTG CTG TGG GAA ATG CAA G	GCT GTA TGA CGA TCG GTG TG
Pfn2	CCG GAC AAA GAG TCA AGG TGG GG	GAC CAA GAC TCT CCC GGC CCT
Pkcb	GAA ACT CGA ACG CAA GGA GA	ACC GGT CGA AGT TTT CAG C
Snca	AAG AAG AGT CTG TTC GCT GGA	AAA GAT GTA TTT TTG CTC CAC ACT T
Phactr1	TCA GAC ATT ATG GAT GGA CCA G	GGA TCT TGG GAC AGG AGG AC
Snap25	GCT CCT CCA CTC TTG CTA CC	CAG CAA GTC AGT GGT GCT TC
Wipf2	ACC ACT GTC CGC TCC TTC T	TCT GGA GCA GGG AAG TCC T
Zdhhc9	ACA CTC TTC TTT GCC TTC GAG T	ATA GCG GCA AAC ACA GGA AT
